# Study of Genetic Association With *DCDC2* and Developmental Dyslexia in Hong Kong Chinese Children

**DOI:** 10.2174/1745017901713010104

**Published:** 2017-08-21

**Authors:** Mary M.Y. Waye, Lim K. Poo, Connie S-H Ho

**Affiliations:** 1 The Nethersole School of Nursing, The Nethersole School of Nursing, The Chinese University of Hong Kong, Hong Kong; 2 Department of Medicine and Therapeutics, The Chinese University of Hong Kong, Hong Kong; 3 Department of Psychology, The University of Hong Kong, Hong Kong

**Keywords:** *DCDC2*, Developmental dyslexia, Genetic association, Sliding window haplotype analysis, Genetic variants, Chinese

## Abstract

**Background:**

Doublecortin domain-containing 2 (DCDC2) is a doublecortin domain-containing gene family member and the doublecortin domain has been demonstrated to bind to tubulin and enhance microtubule polymerization. It has been associated with developmental dyslexia and this protein family member is thought to function in neuronal migration where it may affect the signaling of primary cilia.

**Objectives:**

The objective of the study is to find out if there is any association of genetic variants of DCDC2 with developmental dyslexia in Chinese children from Hong Kong.

**Methods:**

The dyslexic children were diagnosed as developmental dyslexia (DD) using the Hong Kong Test of Specific Learning Difficulties in Reading and Writing (HKT-SpLD) by the Department of Health, Hong Kong. Saliva specimens were collected and their genotypes of DCDC2 were studied by DNA sequencing or TaqMan Real Time PCR Assays.

**Results:**

The most significant marker is rs6940827 which is associated with DD with nominal p-value (0.011). However, this marker did not remain significant after multiple testing corrections and the adjusted p-value from permutation test was 0.1329. Using sliding window haplotype analysis, several haplotypes were found to be nominally associated with DD. The smallest nominal p values was 0.0036 (rs2996452-rs1318700, C-A). However, none of the p values could withstand the multiple testing corrections.

**Conclusion:**

Despite early findings that DCDC2 is a strong candidate for developmental dyslexia and that some of the genetic variants have been linked to brain structure and functions, our findings showed that DCDC2 is not strongly associated with dyslexia.

## 1 INTRODUCTION

The first dyslexia candidate gene *DYX1C1* was reported [1] and confirmed by various groups [2], including our group [3], and it has been shown to play a molecular role in brain development. Knocking down the function of *DYX1C1* using small interfering RNA (siRNA) resulted in disruption of normal neuronal migration in the developing neocortex of embryonic rat, which could be reversed by the concurrent overexpression of *DYX1C1* [4-6]. Disruption of *DYX1C1* also impaired auditory processing and spatial learning in rodent models [7].

Various linkage analyses results showed strong evidences for 6p21.3 in relation to dyslexia [8-13], though some groups reported some difficulties in confirming their results [14] or different results depending on whether the subjects were from UK or USA [15-18]. Deffenbacher *et al.* (2004) [8] used a similar approach by using single nucleotide polymorphism (SNP) markers in the association study. In the first stage, they refined the region to about 3.24Mb by linkage analysis. After that, high dense SNP markers (~21kp apart) across the highly significant region were used. Thirteen SNPs showed significant association with at least one of the phenotypes. The region of these SNPs clustering contains five genes: *VMP, DCDC2, KIAA0319, TTRAP* and *THEM2*.

Thirteen of the seventeen significant associated SNPs were located within *KIAA0319*. Association of KIAA0319 was further confirmed by a study of two large independent UK samples (Oxford and Cardiff) [19]. Harold *et al.* (2006) [19] refined the region by 36 SNPs markers and the region flanking from the first intron to 5’ upstream showed the most significant association with developmental dyslexia (DD). rs3212236 located at 5’ was the most significant marker associated with word choice test (OC-choice), orthographic coding using irregular words (OC-irreg), single-word reading ability (READ) and spelling ability (SPELL) in the Oxford sample. Our group has also confirmed that a common haplotype of KIAA0319 contributes to phonological awareness skill in Chinese children [20]. Recently it has also been reported that brainstem responses as characterized by a reduction in neural discrimination abilities are associated with a higher number of risk alleles of KIAA0319 while no significant association has been found with DCDC2 (rs807724, rs1087266, rs807701, rs793842, rs1091047, rs6922023) and performance in reading and writing [21].

Furthermore, targeted knock down of other dyslexia susceptibility candidate genes (such as *KIAA0319* and *DCDC2*) resulted in similar patterns of neuronal migration [22-24]. Functional analyses of these variations have also been studied indicating that *Kiaa 0319* is expressed during development of mouse and human fetal brain and is involved in neuronal migration for the formation of the cerebral neocortex [22]. Knockdown rat model of *Dcdc2* by RNAi gave different phenotypes in contrast to an earlier study, in that these rats cannot identify specific speech sounds from a continuous train of speech sounds but they did not have problem in discrimination of isolated speech sounds [25]. The mechanism of the effect of *DCDC2* in neurodevelopmental disorder might be due to its function as one of the genes important in the formation of primary cilia, which could lead to determination of visceral asymmetry and brain lateralization [26, 27].

Other genes on chromosome 6p21 region have also been explored. An American group [28, 29]followed up their previous study [30] and refined the 1.5Mb region by a high density marker panel of 147 SNPs. In their results, the strongest association (*p ≤ 0.01*) with discriminant score (DISC) phenotype was shown at the SNPs (rs807724 and rs1087266) of *DCDC2* gene. In particular, reading skills were found to be associated with *DCDC2* in a group of children born in England [31]. Meng’s group also found a 2,445bp deletion polymorphism located in intron 2 also harboring a compound short tandem repeat (STR) polymorphism. The STR comprised of 10 alleles containing variable copy numbers of (GAGAGGAAGGAAA)_n_ and (GGAA)_n_ repeat units (named as “READ1” or “regulatory element associated with dyslexia 1” [32, 33]). By combining the deletion and 10 minor alleles, a significant association was found with homonym choice phenotype [29]. The deletion polymorphism, dbSTS ID no. 808238, encodes multiple copies of PEA3 and NF-ATp sites that are active in brain, and it was hypothesized that large deletion of this region which was found in dyslexics reported by Meng *et al*, would have a profound effect on *DCDC2* function [29]. The deletion present in Chinese occurs at a much higher allele frequency (0.384) than in other populations: US (0.085), German (0.086) and Italy (0.067) [16, 23, 28].

The aim of our study was to find out if there are any association of genetic variants of *DCDC2* with developmental dyslexia in the Chinese children from Hong Kong. To address this question, we use both genotyping with SNPs as well as deletion polymorphisms and in particular the dbSTS ID no.l 808238 that have been described by Meng’s reports.

## 2 MATERIALS AND METHODS

The procedures of subject recruitment, characteristics, and DNA extraction methodology has been reported previously in our earlier reports [3, 20, 34]. In this study, 54 trios aged between 5 and 16 years were used. Subjects were diagnosed as DD cases using the Hong Kong Test of Specific Learning Difficulties in Reading and Writing (HKT-SpLD) [35] and referred by the local education authority, child assessment centres, and a parent association named Hong Kong Association for Specific Learning Disabilities. The HKT-SpLD battery consisted of 12 subtests. The subtests were broken down into three literacy tests, which are Chinese Word Reading (CWR), One-minute Reading (OMR) and Chinese Word Dictation (CWD), and one rapid naming test (DRN), where subjects were asked to name digits. Two subtests involve phonological awareness which is based on testing the subjects’ awareness of onsets (OD) and rhymes (RD) of Chinese syllables, and three were phonological memory subtests, where subjects were asked to repeat orally the syllables presented to them from a disc player, *i.e.* (Word Repetition I (WPI), Non-word Repetition (NWR), Word repetition II (WRII). The final three subtests were tests of orthographic skills. These tests consist of 70 simple Chinese integrated characters and Arabic numbers. Half of them were left/right reversed and the subjects were asked to cross out all items with an incorrect orientation, *i.e.*, Left-Right Reversal (LRR). The subjects were asked to decide characters vs. non-characters, *i.e.*, Lexical Decision (LD) and to identify the correct position of orthographic radicals in Chinese characters, *i.e.*, Radical Position (RP).

These 12 subtests were combined to yield five composite scores in the domains of literacy, phonological awareness, phonological memory, rapid naming and orthographic skills. To be classified as children with dyslexia, their literacy composite score and at least one cognitive composite score had to be at least one standard deviation (SD = 3) below the means (mean = 10) of their respective ages in the HKT-SpLD (cutoff score = 7). Participants in the dyslexic group fulfilled this diagnostic criterion and all of the subjects showed a normal intelligence score on Raven’s Standard Progressive Matrices (with IQs of 85 or above). Saliva samples were first obtained from the participants in the dyslexic group. DNA samples were then extracted from these samples using the OrageneTM DNA self-collection kit following the manufacturer’s instructions (DNA Genotek, Inc., Ottawa, Canada). Permutation test (1000 runs) was used to run multiple testing corrections over all tests performed in single-marker association analyses of categorical DD.

Linkage disequilibrium (LD) was calculated and LD plots were generated using Haploview version 4.1 (http://www.broad.mit.edu/mpg/haploview) [36]. Odds ratio was estimated using the UNPHASED software for discrete analysis (affection status: diagnosed as dyslexia or not). For quantitative traits analysis, additive genetic value (AddVal) was estimated using the UNPHASED software, showing that the value shows the change in expected trait value due to the haplotype of interest relative to the reference haplotype selected. AddVal assumes a normally distributed trait and small deviations from the mean. Gene-gene interactions were analyzed using haplotype-based analysis with the UNPHASED software. The approach compares null hypothesis of equal contributions for all gene combinations in haplotype form sharing the same alleles at the conditioning marker to alternative hypothesis that is differential multiplicative contributions from the test marker. Chi-square tests were used for statistical analyses.

## 3 RESULTS

### 3.1 Association of *DCDC2* with Chinese Dyslexic Children

The most significant marker was rs6940827 associated with DD with nominal p-value (0.011). However, this marker did not remain significant after multiple testing corrections and the adjusted p-value from permutation test was 0.1329 Table (**1**). Using sliding window haplotype analysis, several haplotypes were found to be nominally associated with DD. The smallest nominal p value was 0.0036 (rs2996452-rs1318700, C-A) Table (**2**). Given an ordered set of markers (1, 2....n), sliding windows of overlapping haplotypes were tested in sequence, *i.e.* markers 1-2 were treated as a single haplotype, then markers 2-3 were treated as a single haplotype, followed by markers 3-4, *etc*. Haplotypes of varying sizes (*i.e.* 2-, 3-SNP haplotypes, *etc*.) were assessed. However, none of the p values could withdraw the multiple testing corrections. Quantitative traits analysis was also performed and the nominal significant markers as shown in Table (**3**). Again, none of the markers could withdraw the multiple testing corrections.

### 3.2 Gene-Gene Interaction Analysis

Since a German group has found some interactions between *DCDC2* and KIAA0319 in a large German dyslexia sample [18], we also attempted to find similar interactions. The significant haplotype rs2760157-rs807507 of *KIAA0319* found in our previous study [20] was used for testing gene-gene interaction with *DCDC2*. SNP-haplotype testing between rs2760157-rs807507 and each SNP of *DCDC2* studied was evaluated for DD as well as for each reading related trait. As shown in Table (**4**), there was only a nominal significant gene-gene interaction between *KIAA0319* and *DCDC2*. The associations did not remain significant after permutation tests.

The *DCDC2* deletion polymorphisms were studied by Sanger Dideoxy sequencing, using primers described previously [23, 29]. We have used parents with heterozygous alleles for the calculation of disequilibrium of transmission (QTDT analyses) of the risk allele, and the total number of families used for the calculation was 54 out of a total number of 92 families. There was no significant association of known risk allele with dyslexia status and any subtest of the dyslexic scores, when the deletion risk allele (*i.e.* dbSTS ID no. 808238) was analyzed separately, and when the deletion allele and all remaining minor alleles of dbSTS ID no. 808238 were combined for analyses (*Allele 30). This may be due to the fact that the allele frequency of the deletion polymorphism (listed as allele 14 in Table (**5**) was higher which is very different from those reported for Caucasians.

## 4 DISCUSSION

The prevalence rate of developmental dyslexia in Hong Kong Chinese school-aged children was estimated to be between 9.7% and 12.6% [37], similar to the rate in Caucasian populations [38]. Due to the significant impact of poor reading and writing on performance of students and the strong sense of competition in Chinese education, study of the genetic component of dyslexia is therefore important. Chinese language is known to be substantially different from Western languages, being logographic and morphosyllabic rather than being alphabetic and phonemic [39]. Moreover, orthographic (rather than phonological) deficits were found to be the main problem for Chinese people with dyslexia, in contrast to Caucasians [40]. Functional MRI studies of Chinese people with dyslexia also revealed different biological abnormalities in their brains [41, 42]. Furthermore, altered topological organization of brain structural network showed that dyslexic children exhibited increased local efficiency combined with a tendency of decreased global efficiency and the general prolonged characteristic path length (which might be mainly caused by developmental abnormality of several white matter connections [43]) compared to that of control children, which is somewhat similar to those features observed in congenital amusia Madarin-speaking children of Beijing [44]. Different laboratories have reported conflicting results related to the association of developmental dyslexia with *DCDC2*, our results, as shown below (Table **5**), do not indicate a significant association but rather a nominally significant association only. This could be due to heterogeneity of samples of dyslexia due to different genes responsible for the phenotype or other involvement of unknown gene-environment interactions [45] or some other transcriptional regulatory factors of *DCDC2* [27]. For example, an Italian-Canadian collaboration on *DCDC2* and environmental factors (smoke and miscarriage) underlie attention deficit/hyperactivity disorder traits suggested a potential pleiotropic effect as revealed by a twin study [46]. *DCDC2* gene polymorphisms have been associated with dyslexia in Chinese Uyghur children [47]. Other model system did show a role of *DCDC2* in other aspects of learning which might affect children's language ability. For example, genes targeting *Dcdc2* in the knockdown rat model, impairments of long term memory and visual-spatial performance were reported [48]. Knockdown of DCDC2 was related to a reduction in speech sound discrimination in a continuous stream in rat [21]. Recently it was reported that mutation of the *Dcdc2* leads to enhancement of the glutamatergic synaptic transmission between layer 4 neurons in mouse neocortex [49]. A summary of our findings is provided in (Table **6**).

## CONCLUSION

In conclusion, despite early findings that *DCDC2* is a strong candidate for developmental dyslexia and that some of the genetic variants have been linked to brain structure and functions in several populations [23, 28, 29, 50-54], our findings show that *DCDC2* is not strongly associated with dyslexia in Hong Kong Chinese, consistent with the reports from some other groups [16, 55] and other meta-analyses studies [56]. In contrast to the meta-analyses carried out by Zhong’s group, our findings also do not support their conclusion that rs807701 is associated with developmental dyslexia [57]. However, our results cannot be generalized to all logographical languages as we have only included Hong Kong dyslexic children in this study. Future studies might consider studying FAM65B and CMAHP as new candidate genes in the DYX2 region [9]; further expression studies using immortalized lymphocytes to correlate the expression of *DCDC2* with genotypes, similar to other studies [58] would also be important for further confirmation of our results.

## ETHICAL STATEMENT

This study was approved by the ethical committee of The Chinese University of Hong Kong.

## Figures and Tables

**Table 1:** Single-marker analysis between SNPs of *DCDC2* and categorical DD.

**rs Number**	**SNP**	**Position**	**Location**	**Reference Allele (Frequency)**	**OR** **(95% CI)**	**Nominal p-value**
rs3765502	A/G	24354045	Intron 1	A (0.545)	0.97 (0.67–1.39)	0.8527
rs813227	C/T	24353742	Intron 2	C (0.38)	1.09 (0.73–1.63)	0.6799
rs10498720	G/T	24353436	Intron 2	G (0.178)	1.44 (0.93–2.23)	0.0987
rs6940827	C/T	24351301	Intron 2	C (0.807)	1.82 (1.14–2.9)	0.011*
rs1277351	A/G	24346056	Intron 2	A (0.574)	0.92 (0.64–1.32)	0.641
rs9379655	G/T	24341380	Intron 2	G (0.422)	0.97 (0.68–1.37)	0.8586
rs7770684	C/T	24340212	Intron 2	C (0.848)	0.92 (0.57–1.47)	0.7179
rs9467110	G/T	24321028	Intron 2	G (0.151)	0.94 (0.57–1.54)	0.7995
rs10806987	A/G	24315743	Intron 2	A (0.564)	0.87 (0.61–1.23)	0.4243
rs9379651	A/G	24314900	Intron 2	A (0.391)	0.92 (0.64–1.32)	0.6494
rs11754080	C/T	24284244	Intron 6	C (0.846)	1.14 (0.69–1.87)	0.6113
***rs807701***	A/G	24273791	Intron 7	A (0.801)	1.08 (0.7–1.67)	0.7389
rs870601	C/T	24261060	Intron 7	C (0.803)	1.11 (0.71–1.71)	0.6547
rs9358760	C/T	24249893	Intron 7	C (0.233)	1.18 (0.77–1.82)	0.442
rs1087287	C/G	24237289	Intron 7	C (0.383)	1.06 (0.73–1.54)	0.7738
rs9467080	C/G	24217261	Intron 7	C (0.21)	0.98 (0.63–1.5)	0.9126
rs16888894	C/T	24209886	Intron 7	C (0.415)	0.94 (0.64–1.39)	0.7697
***rs793862***	C/T	24207200	Intron 7	C (0.541)	1.02 (0.7–1.49)	0.923
rs793861	A/T	24206616	Intron 7	A (0.53)	1 (0.69–1.45)	1
rs793850	C/T	24195935	Intron 8	C (0.632)	0.92 (0.62–1.37)	0.6891
rs1343624	C/T	24193138	Intron 8	C (0.598)	0.98 (0.67–1.44)	0.9215
rs3789220	A/G	24189275	Intron 8	A (0.141)	0.79 (0.45–1.37)	0.3956
rs793845	C/T	24188991	Intron 8	C (0.458)	0.98 (0.68–1.43)	0.9244
rs1277190	C/T	24186590	Intron 8	C (0.141)	0.94 (0.56–1.55)	0.7962
rs3804323	C/T	24185739	Intron 8	C (0.802)	0.88 (0.56–1.38)	0.5636
rs2791972	G/T	24185221	Intron 8	G (0.204)	0.89 (0.59–1.36)	0.596
rs2996452	C/T	24180366	Intron 8	C (0.628)	0.76 (0.53–1.1)	0.1434
rs1318700	A/T	24177469	Intron 9	A (0.854)	0.68 (0.41–1.1)	0.1111
rs1936389	G/T	24177457	Intron 9	G (0.41)	0.88 (0.6–1.28)	0.4984

*Adjusted p-value from permutation test is 0.1329.

**Table 2:** Results of the haplotype analysis using 2- or 3-markers sliding window in markers of *DCDC2* gene.

**Haplotypes**		**Frequency**	**Global** **p-values**	**Individual haplotype test**		**Adjusted p-value permutation** **(1000)**
				**OR** **(95% CI)**	**p-values**	
**Sliding window**						
**2-markers**						
rs10498720-rs6940827	G-C	0.188	*0.0185*	1	0.0997	0.2068
	T-C	0.632		1.321 (0.8461 – 2.061)	0.5408	
	T-T	0.180		2.281 (1.255 – 4.148)	0.0116	
rs2996452-rs1318700	C-A	0.475	*0.0276*	1	0.0036	
	C-T	0.137		0.4724 (0.2589 – 0.873)	0.0953	
	T-A	0.371		0.5834 (0.3804 – 0.8947)	0.0922	
	T-T	0.016		0.8063 (0.1407 – 4.62)	0.7442	
**3-markers**						
rs6940827-rs1277351-rs9379655	C-A-G	0.401	0.0251	1	0.6823	0.3227
	C-A-T	0.029		0.5245 (0.1537 – 1.789)	0.2484	
	C-G-T	0.387		0.8443 (0.5539 – 1.287)	0.1061	
	T-A-G	0.013		5.175 (0.6055 - 44.22)	0.0745	
	T-A-T	0.126		1.448 (0.8191 – 7.722)	0.1797	
	T-G-T	0.040		2.675 (0.9268 – 7.722)	0.0435	
**4-markers**						
rs10498720-rs6940827-rs1277351-rs9379655	T-C-A-G	0.398	0.03576	1	0.7079	0.1199
	T-C-G-T	0.216		1.01 (0.624 - 1.634)	0.9921	
	G-C-C-T	0.173		0.7118 (0.4216 - 1.202)	0.0624	
	T-T-A-T	0.126		1.473 (0.8322 - 2.607)	0.181	
	T-T-G-T	0.038		2.466 (0.848 - 7.172)	0.0591	
	T-C-A-T	0.020		0.3875 (0.07633 - 1.967)	0.1575	
	T-T-A-G	0.015		6.143 (0.7291 - 51.76)	0.0537	
rs6940827-rs1277351-rs9379655-rs7770684	C-A-G-C	0.405	0.0089	1	0.6203	
	C-G-T-C	0.379		0.869 (0.5665 - 1.333)	0.2601	
	T-A-T-T	0.117		1.285 (0.7124 - 2.319)	0.3257	
	T-G-T-C	0.040		2.385 (0.8213 - 6.926)	0.0659	
	C-A-T-T	0.024		0.2622 (0.0547 - 1.258)	0.0572	
rs793862-rs793861-rs793850-rs1343624	T-T-C-T	0.390	*0.0383*	1	0.7545	
	C-A-T-C	0.357		0.837 (0.5238 - 1.337)	0.4057	
	C-A-C-C	0.155		1.032 (0.5591 - 1.905)	0.4148	
	T-T-C-C	0.059		1.206 (0.5169 - 2.813)	0.5226	

**Table 3:** Quantitative analysis of *DCDC2* single SNP markers in HKT-SpLD tests. Only markers with nominal p values < 0.05 are shown.

	**Literacy**			**Rapid Naming**	**Phonological Awareness**		**Phonological Memory**			**Orthographic Knowledge**		
	**CWR**	**OMR**	**CWD**	**DRN**	**RD**	**OD**	**WRI**	**NWR**	**WRII**	**LRR**	**LD**	**RP**
rs3789220		0.0443										
rs793845									0.0165			
rs1277190							0.0295		0.0167			
rs2791972									0.0468			
Adjusted P value (permutation x1000)		NS					NS		NS			

**Table 4:** Test of interaction between KIAA0319 and *DCDC2.*

***DCDC2* SNP markers**	**Affection**	***KIAA0319* Haplotype rs2760157-rs807507**											
		**Literacy**			**Rapid Naming**	**Phonological Awareness**		**Phonological Memory**			**Orthographic Knowledge**		
		**CWR**	**OMR**	**CWD**	**DRN**	**RD**	**OD**	**WRI**	**NWR**	**WRII**	**LRR**	**LD**	**RP**
rs3765502	0.4981												
rs813227	0.1955					0.0120							
rs10498720	0.2424		0.0080										
rs6940827	0.6259												
rs1277351	0.3138		0.0160										
rs9379655	0.8371												
rs7770684	0.9912												
rs9467110	0.7079					0.0410							0.0375
rs10806987	0.2613												
rs9379651	0.2071												
rs11754080	0.9245												
rs807701	0.6410												0.0059
rs870601	0.7270												
rs9358760	0.0287	0.0205	0.0258										
rs1087287	0.0084		0.0253			0.0273							
rs9467080	0.7160		0.0440										
rs16888894	0.2402												
rs793862	0.1332							0.0430					
rs793861	0.1044												
rs793850	0.0079												
rs1343624	0.1126												
rs3789220	0.5872												
rs793845	0.3169												
rs1277190	0.2729			0.0020									
rs3804323	0.0113												
rs2791972	0.3055			0.0440		0.0140							
rs2996452	0.6091					0.0400							
rs1318700	0.7176												
rs1936389	0.4401												
Permutation	NS	NS	NS	NS		NS		NS					NS

**Table 5:** Alleles and frequencies of the compound STR, dbSTS ID 808238.

**Allele**	**Repeat unit 1**	**Repeat unit 2**	**SNP1**	**Repeat unit 3**	**Repeat unit 4**	**Repeat unit 5**	Allele Frequency*	Allele Frequency**	Allele Frequency***	**Frequency reported ^^@^**
1	(GAGAGGAAGGAAA)2	(GGAA)7		(GGAA)2	(GGAA)4	(GGGA)2	0.361	0.356	0.481	0.624
2	(GAGAGGAAGGAAA)1	(GGAA)9	DelGAAA	(GGAA)0	(GGAA)4	(GGGA)2	0	0	0	0.003
3	(GAGAGGAAGGAAA)1	(GGAA)6		(GGAA)2	(GGAA)4	(GGGA)2	0	0.005	0	0.06
4	(GAGAGGAAGGAAA)2	(GGAA)6		(GGAA)2	(GGAA)4	(GGGA)2	0.213	0.194	0.183	0.106
5	(GAGAGGAAGGAAA)2	(GGAA)8		(GAA)2	(GGAA)4	(GGGA)2	0.046	0.037	0	0.028
6	(GAGAGGAAGGAAA)2	(GGAA)8		(GGAA)2	(GGAA)3	(GGGA)2	0.009	0.005	0	0.039
7	(GAGAGGAAGGAAA)2	(GGAA)8		(GGAA)1	(GGAA)4	(GGGA)2	0	0	0	0.003
8	(GAGAGGAAGGAAA)2	(GGAA)7	DelGAAA	(GGAA)0	(GGAA)4	(GGGA)2	0	0	0	0.003
9	(GAGAGGAAGGAAA)1	(GGAA)7		(GGAA)2	(GGAA)4	(GGGA)2	0.009	0.014	0.029	0.005
10	(GAGAGGAAGGAAA)2	(GGAA)4		(GGAA)2	(GGAA)4	(GGGA)2	0.009	0.005	0	0.044
11	(GAGAGGAAGGAAA)2	(GGAA)7		(GGAA)2	(GGAA)3	(GGGA)2	0	0	0.01	-
14^#^	x	x	x	x	x	x	0.352	0.384	0.298	0.085

*Frequency among Dyslexic Child of the Dyslexic families **Frequency among Parents of the Dyslexic families ***Frequency among Controls ^^^Allele Types which have been reported in the published paper by Meng *et al.*, 2005. ^@^Frequency among parents of the Colorado Learning Disability Research Center families, reported by Meng *et al.*, 2005. ^#^Allele 14 is the 2,445 bp deletion.

**Table 6:** Summary of results.

**Gene**	**Categorical DD**		**Quantitative Traits**
	**Single marker**	**Haplotype**	**Single marker**
***DCDC2*** ***(SNP)***	Nominal Significant rs6940827	Nominal Significant rs6940827-rs1277351-rs9379655-rs7770684 (most significant) p = 0.0089	Nominal Significant Phonological Memory (WRII) rs793845, rs1277190, rs2791972
***DCDC2 (Deletion)***	Not Significant dbSTS ID 808238	NA	NA
